# Fractures of the Thigh

**Published:** 1828-07

**Authors:** 


14. *Fractures of the Thigh.*—The great number of contrivances, which have been offered to the profession, for the cure of fractures of the thigh bone, prove that a good method has not yet been invented. In the United States no apparatus is in such general use, as that of Desault; but, I believe, few practitioners are satisfied with it, or any of its modifications.

We have ourselves been followers of the great Parisian surgeon; but have encountered the same difficulties with our brethren. The soft parts at each extremity of the limb have, generally, become excoriated; and the patient so restless under that condition, united with his long continuance in an irksome position, that when the time for reunion had arrived, it was almost impossible to maintain the necessary degree of extension.

Considering that, in reference to the proper length of the limb, extension is not required for the first eight or ten days, during which the difficulty of maintaining it, from the fulness of habit and unsubdued muscular power of most patients, at the time of the accident, is far greater than at any other period, we were lately induced, in the case of a boy ten years old, to omit the application of the apparatus, till the second week after the casualty.

When called to the patient, we applied a muslin roller, moderately tight, from the toes to the hip of the affected limb, then placing the patient on his back on a hard bed, we drew up and flexed both knees, over a double inclined plane formed out of bed clothes; and, finally, with another, bound the two legs together throughout their whole length. The patient was left in this position, till the inflammation and fever consequent upon the injury, had subsided, and by abstinence and other antiphlogistics, he was brought into a state of general relaxation. The apparatus of Desault was then applied, and adequate extension effected, with but little difficulty or excoriation. In due time the patient was released from confinement; when his limb was not shortened in the least, nor in any manner deformed.

We would here ask, whether in all the latter stages of the treatment, the limb might not as well be placed over the double inclined plane, and supported by the other, as to be kept in the splints of Desault. As soon as the new osseous matter has acquired a tolerable degree of induration, it is probable, that the weight of the leg, and the support of the other limb, would be quite sufficient to prevent shortening and deformity. Thus the period of permanent extension might, perhaps, be reduced to a single week; and by resorting twice to the method of Pott, and once to that of Desault, the sufferings of the patient would be mitigated, while the chances of decrepitude might be lessened.

Not being professional Surgeons, we throw out these little hints, without pretending to know all that may have been suggested by others; and, in no feeling of anxiety for the manner, in which they may be received by the profession.

